# Notable homologous variation in chromosomal races of the common shrew

**DOI:** 10.3897/CompCytogen.v14i3.54526

**Published:** 2020-07-14

**Authors:** Nina Bulatova

**Affiliations:** 1 A.N. Severtsov Institute of Ecology and Evolution, Russian Academy of Sciences, Moscow 119071, Russia A.N. Severtsov Institute of Ecology and Evolution, Russian Academy of Sciences Moscow Russia

**Keywords:** Chromosome rearrangements (CRs), mammal karyotype, N.I. Vavilov heritage, Robertsonian fusions, *Sorex araneus*

## Abstract

This paper is a review of the rare phenomenon of chromosome intraspecies variation manifested in monobrachial homology series in the comprehensively investigated karyotype of the common shrew *Sorex araneus* Linnaeus, 1758 (Eulipotyphla, Mammalia). The detailed dataset on the account of this mammalian species was drawn from the recently published monograph by Searle et al. (2019) “Shrews, Chromosomes and Speciation”. The parallels to the law of homologous series in variation by Nikolai Vavilov are discussed.

Genetics started in the XXth century with rediscovery of G. Mendel’s hereditary laws, T.H. Morgan’s chromosome theory of heredity and prior evolutionary generalizations of W. Bateson, the author of the term “genetics”. Advances of the first two decades in the emerging field of plant genetics have been promptly consolidated into the law of homologous series in variation by Nikolai Vavilov, who was considered himself to be a student of William Bateson. A concise first presentation of the law idea (Vavilov 1920) in Russian was soon published in English in the Journal of Genetics edited by W. Bateson and R.C. Punnett (Vavilov 1922). Since and till now, homology problems remain in focus of different scientific disciplines exploring homologous variation, from molecular genetics to paleontology and bioinformatics (i.e., Korochkin 1985; Rozhnov 2006; Suslov et al. 2018). For decades, cytogenetic analysis was developing towards the correct assessment of chromosome homology, and today the use of methods of differential staining and, in particular, of chromosome painting makes possible the interspecies comparison on generic and higher taxonomic levels (Ferguson-Smith and Trifonov 2007). This paper aims to review features of homologous chromosomes variation in a mammalian species with one of the best investigated karyotypes, the common shrew *Sorex araneus* Linnaeus, 1758 (Searle et al. 2019). Taking into account the upcoming date of the 100^th^ anniversary of Vavilov’s law, it could be a challenge to examine the variety of chromosomal races of *S. araneus* as the phenomenon of series of homologous variation.

The record of chromosomal variation within the common shrew was recently reviewed in essential details in the monograph “Shrews, Chromosomes and Speciation” which summarized more than the 30-year period of joined multidisciplinary studies of *S. araneus* chromosomal races in Eurasia initiated by the International *Sorex araneus* Cytogenetics Committee, ISACC (Searle et al. 2019). Chapter 5 of this book presents the list of chromosomal races discovered over the whole vast species range of *S. araneus*. As generally, geographic names of 76 chromosomal races were listed in an alphabetical order, accompanied with the diagnostic chromosomal formulas (Bulatova et al. 2019). G-band nomenclature was used for the chromosome identification (Searle et al. 1991) and chromosomal races were defined prioritizing the karyotypic and geographic separation adhering to the ISACC rules (Hausser et al. 1994).

The chromosomes of *S. araneus* are composed of 21 chromosomal arms that can be fused in a variety of combinations in different populations forming an astonishing array of chromosomal races. According to the nomenclature, 21 arms are designated by Latin letters (*a* to *s*) in correspondence with the arrangement in decreasing size from largest to smallest.

In the karyotype of *S. araneus*, chromosomes in pairs appear in either the bi-armed (metacentric) or one-armed (acrocentric) form. Among autosomes, three pairs are always bi-armed and demonstrate stable combination of chromosome arms (*af*, *bc* and *tu*). One other pair is always composed of arms *j* and *l*, but can display individual or population Robertsonian polymorphism appearing in acrocentric or/and metacentric forms (*j*, *l* / *jl*) (Ford and Hamerton 1970).

Sex chromosomes of *S. araneus* have complex origin because of the ancient event of the autosome to sex chromosome translocation: a fusion between the original “true” X (arm *e*) and an autosome (arm *d*). Thus, in females, XX pair is represented by bi-armed (*de*) chromosomes, and in males, by a system of triple sex chromosomes – X(*de*)Y1(“true” Y)Y2(*d*).

Ten other chromosome arms (*g* to *r*, except *j*, *l*) are fused in a variety of combinations which show the remarkable intraspecies polytypic variation. Such arm reshuffling creates a variety of chromosomal races: 37 different combinations of chromosomal arms fused into metacentrics were detected in 76 described chromosomal races (see tables 5.2 and 5.3 in: Bulatova et al. 2019). So, here the set of chromosomes/arms in karyotypes of the *S. araneus* is represented in symbols of the standard nomenclature in Latin letters with variable chromosomes being marked with an asterisk:

*af , bc, de* (XX)/ *d* (Y2), g*, h*, i *, j/l , k*, m*, n*, o*, p* , q*, r*, s (Y1), *tu.*

To analyse the peculiarities of the variable group of chromosome arms, the list of the synoptic table 5.3 from Bulatova et al. (2019) was restructured to follow each chromosome variation in Table 1 here. The acrocentric state and fusion variants were labelled with one and two letters, correspondingly, revealing thus all defined series.

In our analysis, each chromosome series begins with an acrocentric state (for instance, *g*) and accumulates varying fusion combinations with other elements of the variable group (in this case – *gi*, *gk*, *gm*, *go*, *gp*, *gq*, *gr*). That is, from nine possible combinations, two variants of the arm *g* fusions are absent from this series (*gh*, *gn*) – but probably could still be found in nature.

All nine possible fusion variants were realized in two cases, for the arms *o* and *q* (Table 1). Along with aforementioned *g* group (lacking *gh* and *gn*), incomplete series are shown for other arms, namely *h* (-*gh*, *hm*, *hp*, *hr*), *i* (-*in*, *ir*), *k* (-*kn*), *m* (-*hm*), *n* (-*gn*, *in*, *kn*), and *p* (-*hp*), and, correspondingly, for their fusion partners (arms *g*, *h*, *i*, *k*, *m*, *n*, *p*, *r*). It is worth noting that some fusions, for instance *gh* and *hm* absent in the *h* (as well as *g* and *m*) series, were found outside the current list of chromosomal races. These are *hm*, present in an F1 interracial hybrid karyotype due to proposed whole arm reciprocal translocation (WART) (Pavlova et al. 2008), and *gh*, identified in the karyotype of a sibling species, *S. satunini* Ognev, 1922 (Borisov and Orlov 2012). Besides, it seems remarkable that the chromosomes *o* and *q*, most “active” in fusions, are carriers of nucleolus organizing region (NOR), located distally at an acrocentric end (Searle et al. 1991).

Fusions predominate among evolutionary changes of karyotypes in the genus *Sorex* Linnaeus, 1758. Cascades of fusions have happened in the past karyotype evolution of this genus according to refined cytogenetic studies (Biltueva et al. 2011) based on chromosome differential staining (homology of G-banding or R-banding patterns) combined with chromosome painting (chromosome homology revealed via fluorescent *in situ* hybridization). Based on the study of 12 congeneric taxa, the authors proposed an ancestral karyotype of Palearctic *Sorex*, AKPS, consisting of 28 chromosomes (2n = 56). Some of these ancestral elements are homologous to whole *S. araneus* chromosomes, while others to parts of the modern chromosomal arms, indicating that multiple evolutionary fusion events led to the formation of *S. araneus* karyotype. Nine autosomes were completely conserved (*h*, *i*, *m*, *n*, *o*, *p*, *q*, *r* and *tu*), while fusion combinations were defined in nine other chromosomes (*a*, *b* consisting of three parts each: *a*1, *a*2, *a*3 and *b*1, *b*2, *b*3; and those consisting of two parts each – *c*, *d*, *f*, *g*, *j*, *k*, *l*) (Table 2). Subsequent fusions between initially formed acrocentrics shaped two characteristic metacentrics of the *S. araneus* karyotype – *jl* and *bc* (Biltueva et al. 2011). The fusion *jl* is species specific apomorphy, while *bc* is a common fusion for the clade consisting of three sibling species, *S. araneus*, *S. satunini*, *S. antinorii* Bonaparte, 1840 (reviewed by Bulatova et al. 2019). The variants of combination of 10 acrocentrics into 1 to 5 permissible race specific metacentrics are multiplied in series of further fusion combinations of every monobrachial homolog (for instance, *gm* with *hi*, *hk*, *hn* – *hi* with *ko*, *kp*, *kq*, *kr* – *ko* with *nr*, *np*, *nq*; and so on). In such a way, at least 58 distinct karyotypic variants were formed which are attributed to 76 known chromosomal races (note that, sometimes, geographically distant races do not show karyotypic differences).

In this review, numerous series of fusion variations in race specific chromosomes of *Sorex araneus* were considered (Tables 1, 2). It is noteworthy that methodical progress ensures the estimation of chromosome homology within and out of the species limits equally. The data on fully complete and incomplete series are available and allow thinking on and filling in the corresponding gaps. That makes the work in this respect to some extent predictable. It is concluded that homologous series recognition could be in particular demonstrative in cases of interspecies and intraspecies karyotypic variation of the Robertsonian type where it can concur with, for instance, routinely proposed understanding of hybrid origin of metacentric homologs (Bakloushinskaya et al. 2019).

The law of homologous series in chromosome variation may be considered a new notion of comparative cytogenetic studies not only of commemorative (Bulatova 2019) but also of fundamental genetic interests.

**Table 1.** Serial presentation of chromosomal race specific metacentrics (monobrachial homologs) defined in *Sorex araneus*. Asterisks mark the fusions absent* in the list of chromosomal races, and potential** for the race/species karyotypes. A double letter designation is given in the alphabetical order following the standard nomenclature of chromosomes of *S. araneus* (Searle et al. 1991). *o*, *q* – NOR-bearing arms.

*Arm*	*g*	*h*	*i*	*k*	*m*	*n*	*o*	*p*	*q*	*r*	**
***g***		*	*gi*	*gk*	*gm*	*	*go*	*gp*	*gq*	*gr*	gh**
***h***	*		*hi*	*hk*	*	*hn*	*ho*	*	*hq*	*	gh**, hm**
***i***	*gi*	*hi*		*ik*	*im*	*	*io*	*ip*	*iq*	*	
***k***	*gk*	*hk*	*ik*		*km*	*	*ko*	*kp*	*kq*	*kr*	
***m***	*gm*	*	*im*	*km*		*mn*	*mo*	*mp*	*mq*	*mr*	hm**
***n***	*	*hn*	*	*	*mn*		*no*	*np*	*nq*	*nr*	
***o***	*go*	*ho*	*io*	*ko*	*mo*	*no*		*op*	*oq*	*or*	NOR
***p***	*gp*	*	*ip*	*kp*	*mp*	*np*	*op*		*pq*	*pr*	
***q***	*gq*	*hq*	*iq*	*kq*	*mq*	*nq*	*oq*	*pq*		*qr*	NOR
***r***	*gr*	*	*	*kr*	*mr*	*nr*	*or*	*pr*	*qr*		
**Total of 9**	7	5	7	8	8	6	9	8	9	7	

**Table 2.** The chromosomal arms of the common shrew in alphabetical order (top line), without *s* (Y1). Homology to the elements of the *Sorex* ancestral karyotype, AKPS, is indicated in the rows below. Number of ancestral homologous elements that formed present-day arms through past fusions was identified by G-banding and chromosome painting by Biltueva et al. (2011).

a	b	c	d	e	f	g	h	i	j	k	l	m	n	o	p	q	r	tu
a1	b1	c1	d1	e	f1	g1	h	i	j1	k1	l1	m	n	o	p	q	r	tu
a2	b2	c2	d2		f2	g2			j2	k2	l2							
a3	b3																	
